# Hepatocellular Carcinoma With Tumor Thrombus to the Hepatic Veins and the Right Atrium: A Case Report and Review Exploring Various Presentations and Treatment Options

**DOI:** 10.7759/cureus.8405

**Published:** 2020-06-02

**Authors:** Jessica Wassef, Shelley Xu

**Affiliations:** 1 Surgery, Broward Health Medical Center, Fort Lauderdale, USA; 2 General Surgery, Hackensack Meridian Palisades Medical Center, North Bergen, USA; 3 Internal Medicine, Legacy Health, Portland, USA

**Keywords:** hepatocellular carcinoma, tumor thrombus, inferior vena cava, right atrium, hepatitis c, hepatitis b, alcohol, cirrhosis, heart, screening

## Abstract

Hepatocellular carcinoma (HCC) is a leading cause of cancer and cancer-related deaths in the world. Some of the risk factors for the development of HCC include Hepatitis B virus (HBV), Hepatitis C virus (HCV), chronic alcoholism, autoimmune hepatitis, among others. One manifestation of HCC includes tumor thrombus (TT) to the right atrium (RA), which occurs in 0.67-4.1% of patients with HCC. Our case focuses on a unique presentation of HCC with RA TT with initial symptoms of nausea and vomiting without signs of cardiac decompensation or hemodynamic instability. Although there is no definitive treatment for TT to the RA, there are a variety of proven avenues of management of HCC TT to the RA, especially pertaining to patients with adequate liver function.

A 63-year old female with a past medical history of untreated HCV and alcohol abuse with no previously known liver disease or history of liver decompensation, presented with nausea, vomiting, and diarrhea. Initial labs revealed hypovolemic hyponatremia and transaminitis with negative ethanol levels. The model for end-stage liver disease (MELD-Na) score was calculated at 27, and she had a Child-Pugh class C score. Follow up labs were significant for elevated alpha-fetoprotein (AFP). Triple-phase CT of the liver revealed a large liver mass with extension into the RA with TT and necrosis of the liver. An echocardiogram revealed a RA mass versus thrombus. Throughout her hospitalization, she never admitted to cardiac symptoms, including shortness of breath, palpitations, or chest pain. No tachycardia was noted, and her blood pressure remained stable. She was not a candidate for surgery or chemotherapy. The patient declined any heroic measures, and palliative care was consulted for further management. She was transferred to hospice, where she died one week later.

There are numerous etiologies and clinical presentations of HCC with TT to the RA. Its disease course is insidious and may not present as symptomatic until there is a sizable tumor burden. Therefore, treatment options for HCC with TT to the RA are reliant on HCC screening for at-risk populations, early diagnosis, and each individual patient’s baseline liver function.

## Introduction

Hepatocellular carcinoma (HCC) is the sixth leading cause of cancer in the world and the second leading cause of cancer-related deaths in the world [1]. Common risk factors in the United States of America (USA) for HCC include baby boomers born between 1945-1965, men, Hispanic race, genotype three co-infection with hepatitis B virus (HBV) or HIV, metabolic syndrome and/or obesity, excessive alcohol use, family history of HCC, and smoking [2].

In this report, we focus on a rare manifestation of HCC, which is tumor thrombus (TT) to the right atrium (RA). This is most commonly due to liver cirrhosis, but can also be caused by inflammatory abdominal processes, such as pancreatitis, and occurs in 0.67-4.1% of patients with HCC [3,4]. Notably, 20% of patients who develop HCC have non-cirrhotic livers, while almost all HCC with TT cases are due to cirrhotic livers [1,3]. Although HCC is prone to hematogenous invasion, it most commonly spreads to the lungs, lymph nodes and adrenal glands, and an extension to the heart is rare [3]. The disease process of HCC with TT to the RA is insidious and can present with many different symptoms that reflect signs of cardiac and/or liver decompensation [3,5-9]. Although there is no gold standard treatment for HCC with TT to the RA, evidence shows patients with adequate liver function may benefit from surgery, radiation, and chemotherapy. As HCC therapy relies on preserved liver function and compliance with screening, early management of the liver disease is vital. 

## Case presentation

A 63-year old Caucasian female initially presented with persistent nausea and non-bloody, non-bilious vomiting for two weeks. She endorsed bloating and two episodes of non-bloody watery diarrhea. Her past medical history included untreated hepatitis C virus (HCV), hypertension, lung nodules, alcohol abuse, including a daily alcohol intake of one liter, and a history of intravenous drug use. She also had no previously known liver disease with no history of liver decompensation, including variceal bleeding, hepatic encephalopathy, or spontaneous bacterial peritonitis. On exam, the patient appeared jaundiced and was found to have a distended abdomen, moderate ascites, and a palpable lobulated liver with 2+ pitting edema on the bilateral lower extremities. No spider angiomas or jugular venous distension were appreciated. Initial labs revealed hypovolemic hyponatremia, aspartate aminotransferase (AST) of 93 U/L, international normalized ratio (INR) 1.4, albumin 3.2 g/dL, and a total bilirubin level of 4.4 mg/dL. A model for end-stage liver disease (MELD-Na) score was calculated at 27, and she had a Child-Pugh class C score. She had a hepatitis C virus (HCV) RNA level of 1075 IU/mL.

**Figure 1:**
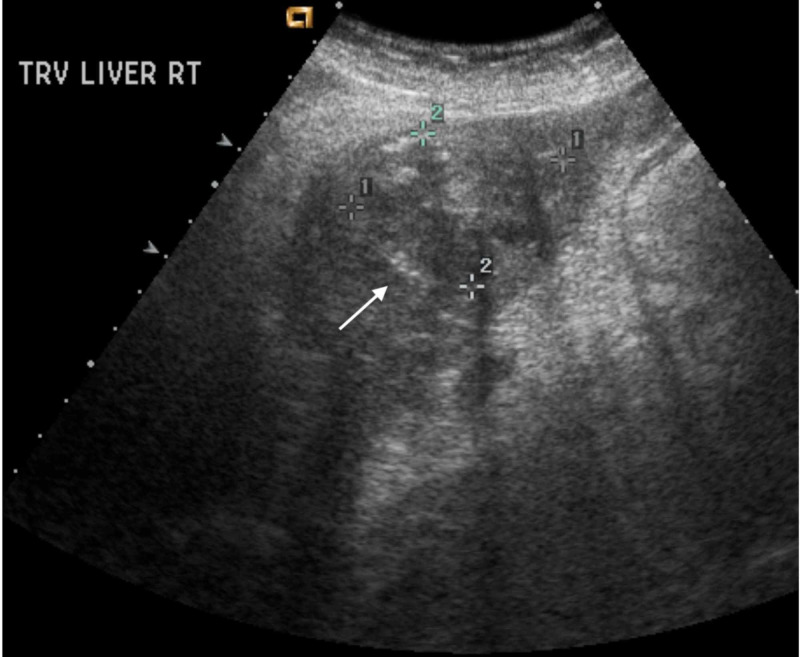
Liver Ultrasound Liver Ultrasound revealed a 4.9 cm x 3.6 cm x 4.2 cm mass in the left lobe with a small amount of perihepatic ascites.

**Figure 2:**
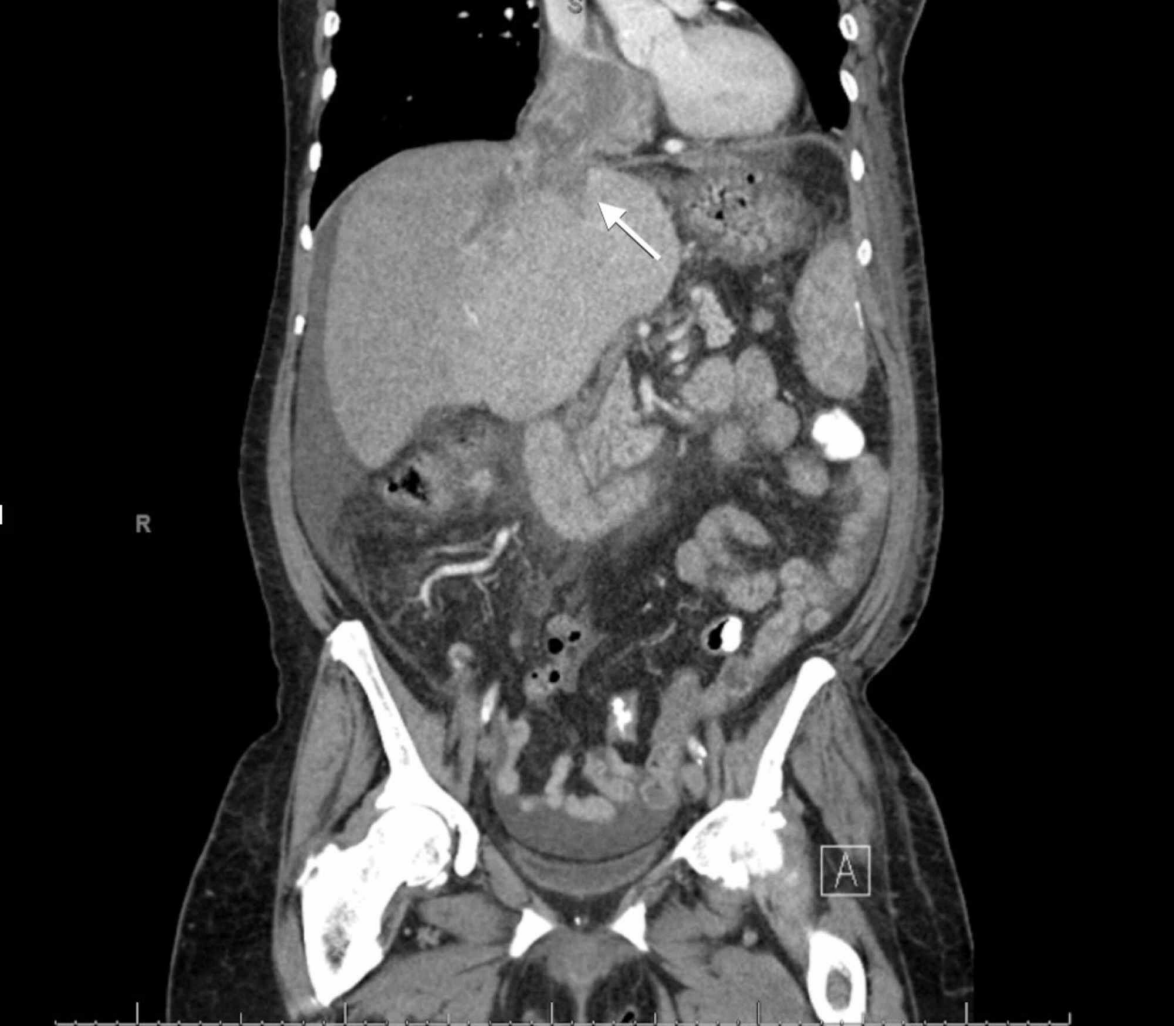
Triple-Phase Liver CT - Coronal view Coronal view of triple-phase liver CT scan revealed a tumor thrombus occupying the middle and left hepatic vein that partly extended into the right atrium.

**Figure 3:**
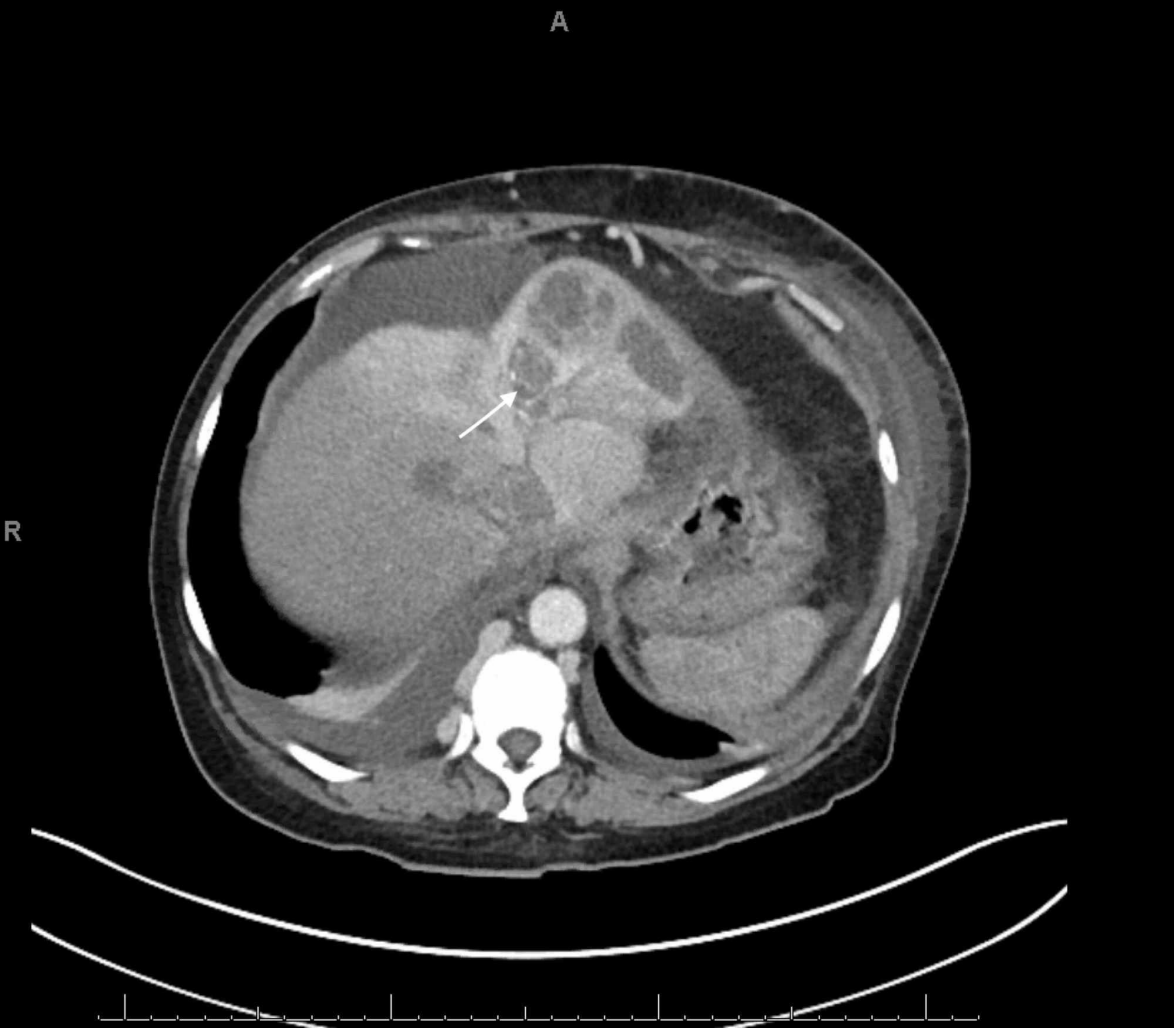
Triple-Phase Liver CT - Transverse section of the Liver Triple-phase liver CT revealed a mass occupying the left hepatic lobe with necrosis of the liver.

**Figure 4:**
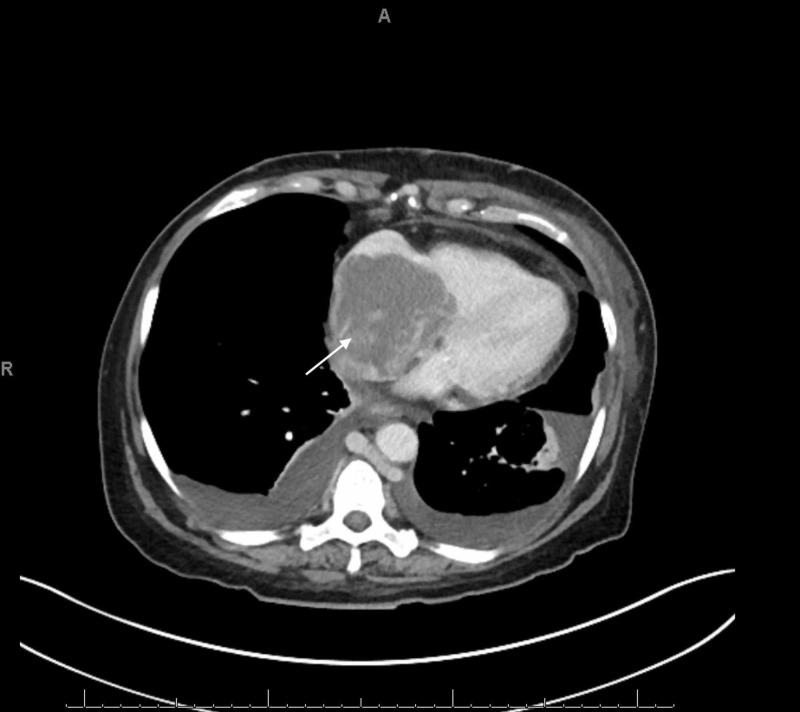
Triple-Phase Liver CT - Transverse Section of the Right Atrium Transverse view of triple-phase liver CT revealed a mass in the RA with tricuspid involvement and its own vascular supply.

**Figure 5:**
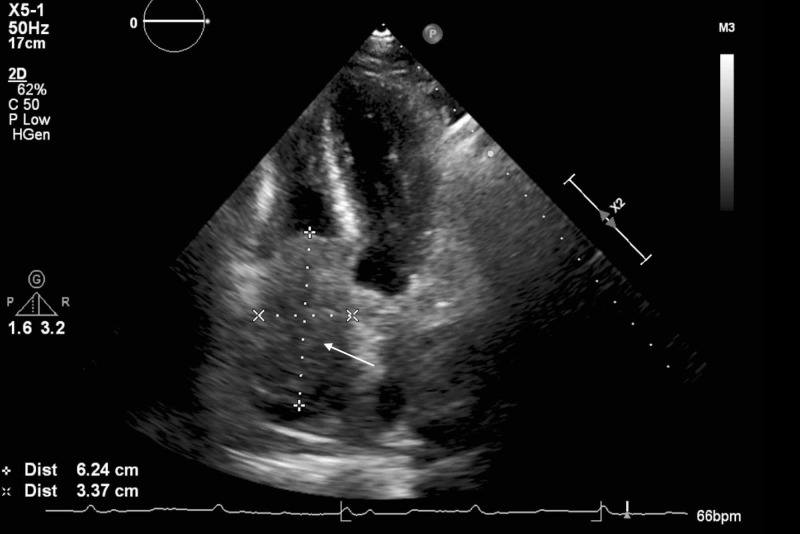
Echocardiogram Echocardiogram revealed the 6 cm x 3 cm mass or thrombus occupying the right atrium with tricuspid involvement. There is no visible extension into the superior vena cava.

An abdominal x-ray showed a non-obstructive bowel pattern. A computed tomography (CT) of the abdomen suggested a mass in the left lobe of the liver and was followed by an ultrasound (US) (Figure 1), which revealed a 4.9 cm x 3.6 cm x 4.2 cm mass in the left lobe of the liver with perihepatic ascites. Follow up labs were significant for elevated alpha-fetoprotein (AFP) at 6160.2 ng/mL (reference range: 0.0 to 9.0 ng/mL) and elevated lactate dehydrogenase at 576 U/L. Follow up with a triple-phase liver CT (Figures 2-4) revealed a large liver mass with extension into the RA with TT and necrosis of the liver. An echocardiogram (Figure 5) revealed a 6 cm x 3 cm RA mass versus thrombus.

The patient never presented with any cardiac symptoms during her stay at the hospital. Throughout her hospitalization, she denied shortness of breath, chest pain, or palpitations. No tachycardia was noted, and her blood pressure remained stable.

She was seen by gastroenterology, hepatobiliary surgery, and oncology. All teams agreed that she was not a candidate for surgery or chemotherapy due to her advanced staging, thrombocytopenia, and coagulopathy. The patient was offered Sorafenib, which she declined, and palliative care was consulted for further management. She was transferred to hospice, where she died one week later.

## Discussion

Review of cases of HCC tumor thrombus to the right atrium

In reviewing six other case reports of HCC with RA TT without intervention (Table 1), our case presentation specifically focuses on our patient’s unique presentation of HCC with RA TT with initial symptoms including nausea and vomiting without signs of cardiac decompensation or hemodynamic instability.

**Table 1:** Review of cases of HCC with Tumor Thrombus to the Right Atrium Abbreviations: HCV, hepatitis C virus; HBV, hepatitis B virus; TACE, trans-arterial chemoembolization; HCC, hepatocellular carcinoma.

Citation #	First Author	Article	Year	Etiology	Initial Symptoms	Intervention
[3]	Steinberg	Advanced hepatocellular carcinoma with subtotal occlusion of the inferior vena cava and a right atrial mass	2013	Unknown	Generalized edema	Palliative care
[5]	Lourenço	Hepatocellular carcinoma presenting with Budd-Chiari syndrome, right atrial thrombus and pulmonary emboli.	2017	HCV	Right upper quadrant pain, dyspnea, abdominal distension	Palliative care
[6]	Numan	Hepatocellular carcinoma with tumor thrombus extending from the portal vein to the right atrium.	2019	HCV	Melena	Palliative care
[7]	Albakr	A large right atrial mass in a patient with hepatocellular carcinoma: Case report and literature review.	2014	HBV	Cachexia	Sorafenib for two months with poor tolerance
[8]	Huang	Hepatocellular carcinoma with inferior vena caval and right atrial tumor thrombi and massive pulmonary artery embolism: A case report.	2016	HBV	Dyspnea	Palliative care
[9]	Sempokuya	Right atrium invasion of tumor thrombus from hepatocellular carcinoma incidentally found on transthoracic echocardiogram.	2018	Autoimmune hepatitis	Cachexia, abdominal distension, dyspnea and edema	Sorafenib, TACE, and radiofrequency ablation, for HCC, however, did not receive further intervention once tumor thrombus was diagnosed.

The other published case studies revealed various presentations of HCC with RA TT including cachexia, generalized edema, right upper quadrant pain and dyspnea secondary to Budd-Chiari syndrome, dyspnea secondary to pulmonary embolism, melena due to esophageal varices, and a combination of cachexia, abdominal distension, dyspnea and edema [3,5-9]. It is, therefore, important to emphasize the wide variety of presentations of HCC with RA TT, as none of these patients presented with primary cardiac symptoms that could otherwise be explained by liver decompensation. 

Our patient also had the unique etiology of HCC with RA TT likely caused by HCV in conjunction with excessive alcohol consumption. Among the six cases we reviewed, two cases were due to HCV as seen in our patient, two cases were due to HBV, one case was due to autoimmune hepatitis, and one case was of unknown etiology [3,5-9]. Patients with untreated hepatitis C (HCV) infection, as seen in our patient, have a 15 to 20 fold higher risk of HCC compared with the general population [2]. History of alcohol abuse, especially in women, also increases the risk of HCC as even a moderate intake of alcohol in women increases the risk of HCC more than in men. In addition, alcohol use also exhibits a strong synergistic effect with HCV in increasing the risk of developing HCC, which is exacerbated further by alcohol abuse as a female [2]. 

Similar to our patient, these cases received little to no intervention after the final diagnosis of their disease. One patient refused treatment, one patient started Sorafenib for two months with poor tolerance, and three of the patients received palliative care [3,5-8]. One patient had previously tried Sorafenib, radiofrequency ablation (RFA), and trans-arterial chemoembolization (TACE) for her HCC, but did not receive intervention once the tumor thrombus was diagnosed [9]. These cases show how limited the treatment options become once HCC advances to TT and emphasize the importance of early detection of HCC.

Tumor thrombus in non-cirrhotic patients

Portal venous tumor thrombus (PVTT) can occur both in patients with cirrhosis and without cirrhosis. However, almost all cases of PVTT exist in patients with previous liver cirrhosis or at least one traditional risk factor for HCC [3]. In non-cirrhotic patients without HCC, PVTT is often due to inflammatory abdominal conditions, such as pancreatitis and diverticulitis [10]. PVTT can also form due to intra-abdominal malignancies and liver metastasis, such as colorectal cancer [10]. In Western countries, it is very rare to see HCC without cirrhosis, but it has been observed with patients with large cell dysplasia or iron overload [11]. Injuries to the portal venous system have also been found to cause PVTT [12]. However, studies have shown that 4.1% of patients with HCC were found to have RA TT upon autopsy without previous diagnosis [4].

Review of interventions

There is no definitive treatment for HCC leading to tumor thrombus in the RA. In patients with good hepatic function and those who meet Child-Pugh class A criteria, studies have shown that surgical and non-surgical interventions can be successful. For minimal RA TT invasion, partial hepatectomies with thrombectomy, and total hepatic vascular exclusion (THVE) can be performed. This process involves reducing the liver to mobilize the TT downward from the RA into the IVC and clamping the IVC superior to the thrombus, followed by THVE and removal of the thrombus followed by partial hepatectomy [13]. 

In situations with more invasive RA TT in which the thrombus cannot be mobilized into the IVC, THVE with cardiopulmonary bypass (CPB) has been studied to be effective. CPB utilizes an outside circuit that navigates venous blood to a reservoir, which is then oxygenated and pumped back into the body. In a study by Wakayama et al. studying surgical intervention in 13 patients with HCC with TT to the IVC and RA, five of the patients who underwent a curative resection experienced a recurrence with a total median survival of 15.3 months for patients with an IVC TT and 11.2 months in patients with an IVC TT extending to the RA [14]. Another study by Inoue et al. reviewed this intervention in 19 cases and found that 21.1% had a survival of more than two years, with 38% not showing any recurrence of HCC by the termination of the study and a median survival of 11 months [15]. 

Radiation therapy has also been practiced in patients with HCC with TT to the RA. Although surgery has been proven to be superior in efficacy to radiation therapy, radiation therapy has shown to be overall better tolerated in patients and has moderate tumor response [12-15]. Tumors treated with external beam radiotherapy may progress more slowly in those with intrahepatic tumor size less than 10cm [16]. It may also be beneficial in cases of recurrent HCC with TT. A study by Lou et al. found TT resolution in 22.7% of patients with recurrent HCC with either TT in the IVC or both the IVC and RA, a median survival of 11 months in those with TT to the IVC, and a median survival of eight months in those with TT to the IVC and RA using hypofractionated radiotherapy [17]. RFA and TACE are better used in combination with the previously listed interventions, although they do provide some relief independently and prolong survival [18]. In a study by Duan et al., in which they used RFA and TACE followed by TACE on new intrahepatic lesions, they found a median survival of 21 months with a three-year survival of 27.3% [18].

Evaluating liver function and cirrhosis severity when electing screening and treatment options for HCC

In considering treatment options, it is important to consider the patient’s liver function. One commonly used classification system in patients with liver disease is the MELD-Na score, which calculates a three-month mortality rate based on kidney function, bilirubin, INR, and sodium. Our patient had an initial MELD-Na score of 27, possibly contributed by untreated HCV, chronic alcoholism, and HCC. A MELD-Na score of 27 indicates an estimated three-month mortality rate of 19.6%. Any patient with a MELD-Na score greater than 10 should be referred to a hepatologist. Another common assessment of liver function is the Child-Pugh score, which assesses cirrhosis severity by creating a score based on total bilirubin, albumin, prothrombin time, INR, ascites, and degree of encephalopathy. Treatment for HCC with TT to the RA may be dependent on the class the patient falls in. All of the patients treated with surgical intervention in the previous studies had a Child-Pugh class A score [13,14,17]. Patients with Child-Pugh class B score were only treated for recurrence with radiation and had an overall poor prognosis [18]. None of the patients in the prior studies outlining intervention who were treated with surgery, radiation, or TACE treatments had a Child-Pugh class C score, which has a predicted life expectancy of one to three years [13,14,17]. Chronic alcohol use and HCV are important risk factors that may lead to cirrhosis and her Child-Pugh score. Due to this patient’s poor liver function and advanced staging of HCC, she had no options available to her except Sorafenib, a second-line multikinase inhibitor proven to prolong survival by one to three months, which she denied [19]. 

HCC screening guidelines

Screening and prevention of HCV in the USA should be a public health priority, as HCV continues to be the leading cause of HCC in the USA [2]. Screening recommendations vary by region and association. The American Association for Study of Liver Disease (AASLD) recommends screening cirrhotic patients with ultrasound and optional AFP every six months. However, it is not recommended to continue to screen patients with Child-Pugh class C unless they are on the transplant list due to poor prognosis. The Asian Pacific Association for the Study of the Liver (APASL-2017) recommends using both AFP and US every six months. The Japan Society of Hepatology (JSH-2015) recommends screening with AFP, protein induced by vitamin K absence or antagonist II (PIVKA -II), and AFP L3 with ultrasound (US) every three to four months with optional multi-detector computed tomography (MDCT) or MRI every six to twelve months for patients in the extremely high-risk category. The extremely high-risk category is defined as patients with cirrhosis secondary to HBV or HCV. For patients in the high-risk category, defined as patients with chronic HBV or HCV and patients with non-viral cirrhosis, they recommend screening with AFP, PIVKA-II, and AFP L3 and US every six months for patients without MDCT or MRI [10,20]. 

## Conclusions

Ultimately, this case highlights the differing etiologies and presentations for HCC with TT to the RA. Our patient’s unique presentation of nausea and vomiting as her primary symptom reveals how variable and insidious this disease can be. Our case report was limited due to lack of intervention and inability to evaluate the extent of metastasis to her regional lymph nodes and distant organs due to her rapid decompensation leading to death a few weeks after diagnosis. Strengths in this study include the evaluation of the extent of the tumor thrombus with CT and an echocardiogram. We were also able to find a possible etiology based on her past medical history and understand the extent of her declining liver function based on her risk factors and laboratory results. Our patient’s attributable risk factors to the development of her HCC with TT to the RA include alcohol abuse and HCV infection. Different screening options are available and involve a combination of imaging and serum markers to monitor for HCC. Few treatments are available in advanced HCC, but surgical intervention should be first-line. Based on our patient’s case and previously published presentations, we have seen that it is imperative for HCC with TT to the RA to be screened and diagnosed early for optimal treatment options. Assessing liver function is paramount in deciding the proper treatment course, and the number of treatment options available decreases in tandem with a decrease in liver function.
